# Development of a Real Time Sparse Non-Negative Matrix Factorization Module for Cochlear Implants by Using xPC Target

**DOI:** 10.3390/s131013861

**Published:** 2013-10-14

**Authors:** Hongmei Hu, Agamemnon Krasoulis, Mark Lutman, Stefan Bleeck

**Affiliations:** 1 Institute of Sound and Vibration Research, University of Southampton, Southampton SO17 1BJ, UK; E-Mails: agamemnon.krasoulis@gmail.com (A.K.); mel@isvr.soton.ac.uk (M.L.); bleeck@gmail.com (S.B.); 2 Department of Mechanical Engineering, Jiangsu University, Zhenjiang 212013, China; 3 Medical Physics, University of Oldenburg and Cluster of Excellence Hearing4all 26129, Germany; 4 School of Informatics, University of Edinburgh, Edinburgh EH8 9AB, UK

**Keywords:** cochlear implants, non-negative matrix factorization, speech enhancement, vocoder, xPC Target, real-time system

## Abstract

Cochlear implants (CIS) require efficient speech processing to maximize information transmission to the brain, especially in noise. A novel CI processing strategy was proposed in our previous studies, in which sparsity-constrained non-negative matrix factorization (NMF) was applied to the envelope matrix in order to improve the CI performance in noisy environments. It showed that the algorithm needs to be adaptive, rather than fixed, in order to adjust to acoustical conditions and individual characteristics. Here, we explore the benefit of a system that allows the user to adjust the signal processing in real time according to their individual listening needs and their individual hearing capabilities. In this system, which is based on MATLAB^®^, SIMULINK^®^ and the xPC Target™ environment, the input/outupt (I/O) boards are interfaced between the SIMULINK blocks and the CI stimulation system, such that the output can be controlled successfully in the manner of a hardware-in-the-loop (HIL) simulation, hence offering a convenient way to implement a real time signal processing module that does not require any low level language. The sparsity constrained parameter of the algorithm was adapted online subjectively during an experiment with normal-hearing subjects and noise vocoded speech simulation. Results show that subjects chose different parameter values according to their own intelligibility preferences, indicating that adaptive real time algorithms are beneficial to fully explore subjective preferences. We conclude that the adaptive real time systems are beneficial for the experimental design, and such systems allow one to conduct psychophysical experiments with high ecological validity.

## 1. Introduction

Various speech processing algorithms have been proposed in the literature to reduce the background noise for different applications [1–12]. Most signal processing algorithms need to be adaptive rather than fixed, in order to adjust to (a) acoustical conditions and (b) individual characteristics (e.g., different characteristics of hearing capability or pathology). Usually, signal processing algorithms are fixed or may be first adjusted for each user and then fixed. We wished to explore the benefit of a system that potentially allows the user to adjust the processing according to their individual listening needs at a particular time and their individual hearing capabilities. None of these can be predicted ahead with our current state of knowledge, so a real-time adaptive system is needed. In order to explore this idea, we needed to first implement a real-time system and, then, evaluate whether it was potentially beneficial. In this paper, a real time non-negative matrix factorization (NMF)-based speech processing strategy for cochlear implants (CIS) will be implemented and evaluated to explore this idea.

Cochlear implants (CIS) are electrical devices that can restore partial hearing loss to the profoundly deaf. The main principle of CIs is to stimulate the auditory nerve via electrodes that are surgically inserted into the inner ear. With the development of new speech processors and algorithms, CI users benefit more and more from CIS [13]. However, the average speech perception performance of CI users decreases dramatically in the presence of background noise [6,14,15]. Some previous studies showed that statistical model-based speech processing algorithms can improve the speech intelligibility for CI users by reducing the redundancy in noisy speech [16–19]. Recently, non-negative matrix factorization (NMF) [20,21] has been applied successfully at the intersection of many scientific and engineering disciplines, such as image processing, speech processing and pattern classification [22–36]. Motivated by the non-negativity of the envelopes of the CI channels, a novel coding strategy based on sparse constrained NMF [37] was proposed as an alternative method to improve the performance of CIS, especially in noisy environments, by controlling the sparseness of the reconstructed signal [38,39]. This was achieved by making use of a basic NMF method with a sparseness constraint, mainly due to its low computational complexity, always bearing in mind the need for an envisaged real-time implementation.

The first aim of this paper is to implement this coding strategy in real time. Currently, there are several real-time CI research platforms, such as the personal digital assistant (PDA)-based real-time speech processing research platform described in [40] and the xPC Target-based CI research platform used in Cochlear ™ [41]. We present an alternative implementation that allows one to evaluate new algorithms for CI speech processing, specifically, a new implementation of the sparsity-constrained NMF module.

The system is based on MathWorks^®^ xPC Target, and provides a solution for prototyping, testing and deploying real-time systems using standard PC hardware in the manner of hardware-in-the-loop (HIL) simulation [42]. By supporting various standard I/O boards with an extensive I/O device driver library, any need for developing custom interface codes can be avoided.

In our rapid host-target environment, a desktop or laptop computer is used as the host PC. The host PC runs the following software packages: MATLAB^®^, SIMULINK^®^, SIMULINK Coder™ (formerly Real Time Workshop^®^), xPC Target and a C/C++ compiler. The I/O boards are interfaced between the SIMULINK blocks and the application hardware system, *i.e.*, a CI stimulation system. After creating the SIMULINK model, executable code is generated with the SIMULINK Coder and the C/C++ compiler, which is then downloaded from the host PC to the target PC running the xPC Target real-time kernel. The biggest advantage of the platform is that user-specific parameters can be tuned in real time.

The second aim of this paper is to investigate the hypothesis that most signal processing algorithms (such as those based on NMF) need to be adaptive rather than fixed according to different acoustical conditions and individual characteristics. We hypothesized specifically that (i) listeners in general would prefer different settings for different listening conditions (different signal-to-noise ratio (SNR)) and (ii) not all listeners would choose the same settings for any given listening condition. We assume that it is desirable to implement solutions that include suitable real-time adjustment that is either controlled by the listener or, possibly, in future, by a smart algorithm. Such solutions offer a much improved ecologically valid way of experimenting compared to traditional fixed stimuli approaches.

The remainder of the paper is organized as follows: the sparseness constrained NMF algorithm is introduced in Section 2. The sparse NMF speech processing strategy is adapted to CIS in Section 3. The hardware and software used in the real-time implementation are presented in Section 4. Finally, the conclusions of the study are given in Section 5.

## 2. Sparse NMF Strategy for CIs

Given a non-negative input matrix, **Z**, NMF is a method to factorize **Z** into a basis matrix, **W**, and the corresponding component matrix, **H**, so that **Z** ≈ **WH**. To do the factorization, a cost function, *D*(**Z**‖**WH**), is usually defined and minimized. Several possibilities have been suggested for defining the cost function and for performing the consequent minimization [24,25,31]. In this paper, an Euclidean distance -based NMF (EUC**-**NMF), where the square Euclidean distance $D(Z∥WH)=\frac{1}{2}{\left\|Z-WH\right\|}_{2}^{2}$ is used as the cost function, which is equivalent to the Maximum Likelihood (ML) estimation of **W** and **H** in additive independent identically distributed (i.i.d.) Gaussian noise. Since the basic NMF allows a large degree of freedom, different types of regularization have been used in the literature to derive meaningful factorizations for a specific application. In general notation, the following minimization is performed: $\left[\hat{W,}\hat{H}\right]=\arg\underset{W,H}{\text{min}}\left[D(Z∥WH)+f(W)+g(H)\right]$ where *f*(**W**) and *g*(**H**) are regularity functions for the basis matrix, **W**, and the component matrix, **H**. The most common regularization is motivated by the sparseness of the signal [27,28,43,44] and the correlation of the signal overtime [32,44].

## 2.1. Sparseness-Constrained NMF

The sparseness constrained NMF used in our paper was proposed in [43] and improved in [45]. Accordingly, the sparseness constraint used here is directly controlled by the number of nonzero elements. In our solution, the Euclidean cost function was combined with a *L*_1_—regularized least squares sparseness penalty function through a least absolute shrinkage and selection operator (LASSO) framework [37,43]. Furthermore, an additional sparseness constraint was applied to explicitly control the sparsity of the NMF component matrix, **H**, and the optimization algorithm proposed by Hoyer [37,43] was applied to obtain the non-negative matrices, **W** and **H**.

In our real-time CI implementation, **Z** is the envelope of CI-channels in multiple frequency bands, named *envelopegram* here. The sparse NMF algorithm was applied to the *envelopegram* of the input signal using a block by block batch processing by buffering a fixed number of *M* continuous frames in each channel. Let *z*(*t*) denote the measured noisy speech signal, with *t* being the discrete time index and *z_i,j_* the envelope-time bin in the *i^th^* channel of the *j^th^* frame, which is calculated by weighting and summating the short time Fourier transform (STFT) spectrum according to the advanced combination encoder (ACE) strategy [46]. **Z** is an *N* × *M envelopegram*, which contains *N* = 22 channel envelope bins in each column and *M* = 10 frames in each analysis block, which is the same as the one used in [18] and is short enough to allow for real-time implementation. Hence, given the non-negative envelope matrix, **Z**, we aim to obtain the basis matrix, **W**, and component matrix, **H**, such that:

(1) $$D(Z∥WH)=\frac{1}{2}∥Z-WH{∥}_{2}^{2}+\lambda g(H)$$

is minimized, under the constraints that the elements of the factorized matrices are non-negative, *i.e.*, ∀*_i,j,k_* : *w_ik_* ≥ 0, *h_kj_* ≥ 0, λ ≥ 0, where *K* is the component number, **w***_i_* denotes the *i^th^* column of $\begin{array}{l} \text{W},\text{W}={\left[\begin{array}{ccc} w_{11} & \cdots & w_{1K}\\ \vdots & \ddots & \vdots \\ w_{N1} & \cdots & w_{NK} \end{array}\right]}_{N\times K} \end{array}$, $\begin{array}{l} \text{H}={\left[\begin{array}{ccc} h_{11} & \cdots & h_{1M}\\ \vdots & \ddots & \vdots \\ h_{K1} & \cdots & h_{KM} \end{array}\right]}_{K\times M} \end{array}$, $g(\text{H})=\overset{K}{\underset{k=1}{\text{∑}}}\overset{M}{\underset{j=1}{\text{∑}}}h_{kj}$ and λ is the sparsity constraint parameter that controls the level of sparsity.

An iterative algorithm, as proposed by Hoyer [37,43], was implemented to minimize the cost function in Equation (1), in which the basis matrix, **W**, and the component matrix, **H**, are updated by gradient descent and multiplicative update rules, respectively. In our real-time implementation, the buffer length was set to *M* = 10 frames [19,38]. The systematic delay caused by buffering (considering a frame length of 8 ms, and 75% overlap) was around 20 ms. This constitutes an acceptable delay, which is not perceived by the CI listener. The total delay imposed by the algorithm is the sum of the buffering time and processing time for each block. The algorithm was implemented and embedded in the same real-time CI research platform as in [41], which was provided by *Cochlear*™. The choice of *K* is important, as it has to be a compromise between computational costs and speech quality. We performed informal listening tests with *K* values ranging from 5 to 20 and decided that the perceived intelligibility difference does not justify the increased computational costs. We therefore initially set *K* = 5 and will investigate the consequences of this trade-off further in future.

The other important parameter, the sparsity constraint parameter λ in Equation (1), controls the level of sparsity as a compromise between the NMF approximation and sparsity. Because it is not possible to determine an optimal value from the first principles, we developed a two-step parameter selection procedure and evaluated it in detail in [38]. This procedure works in two stages combining objective measurements with subjective experiments: in the first stage, various objective measurements are used to select a range of possible λ values; then, in the second stage, the final value of λ is determined in subjective experiments (refer to [38] for more detail). The single sparsity parameter is an attractive feature for the overall system, because it is an explicit parameter that can easily be tuned by individual users based on their preference to achieve an optimum combination of speech perception performance and speech quality.

## 2.2. Sparse NMF Strategy for CIs

**Figure 1.**
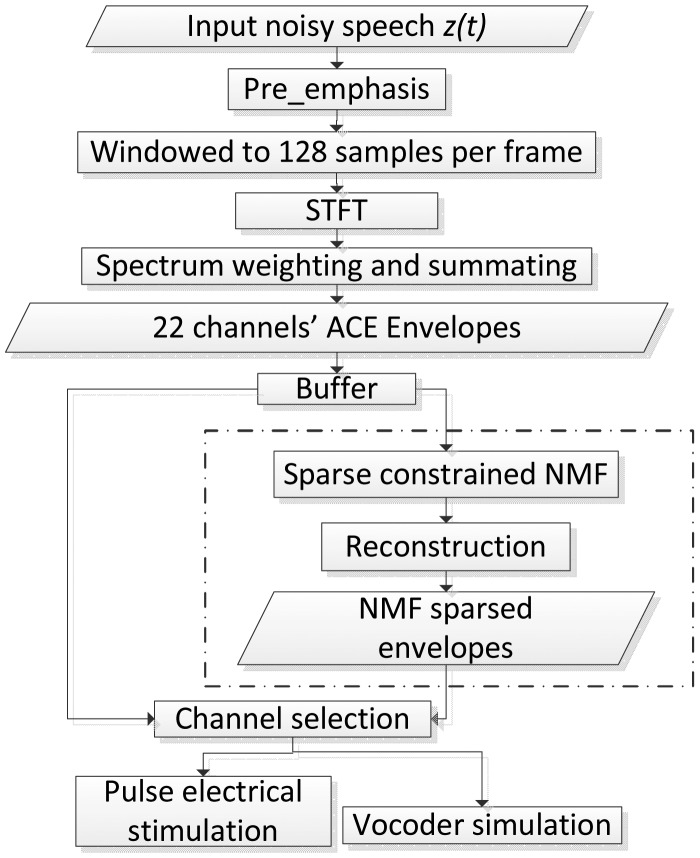
Advanced combination encoder (ACE) strategy and the proposed sparse constrained non-negative matrix factorization (NMF) strategy.

Figure 1 shows the flow chart of the sparse NMF algorithm. The first steps are identical to the standard ACE strategy. The blocks in the dashed frame (‘sparse constrained NMF’, ‘reconstruction’ and ‘sparse NMF processed envelopes’) indicate the modifications in the proposed strategy compared to ACE. The pre-emphasis filter attenuates low frequencies and amplifies high frequencies to compensate for the −6 dB/octave natural slope in the long-term speech spectrum. It emphasizes, for example, low-energy, high-frequency consonants against high-energy, low-frequency vowels. After transforming the input speech signal into a spectrogram, the 22-channel *envelopegram* is extracted by summing the power of the frequency bins within each band. The sparse NMF algorithm is then applied to the *envelopegram* on a block by block basis, by buffering a certain number of continuous frames in each channel. The envelopes are then reconstructed from the modified sparse NMF components [39]. Finally, appropriate channels are selected in order to either stimulate a real CI or to drive a vocoder simulation [47], which can be used in experiments with normal hearing (NH) listeners.

## 2.3. Simulation Results

For the purpose of demonstration, a single word ‘Din’ from the same speech database as in [48] is used. NMF is applied to the whole *envelopegram* with a dimension of 22 ∗ *T*, where *T* is the number of the short-time frames of the word ‘Din’. In this example, the sample rate is *f_s_* = 16 kHz, the length of the word is *L* samples, then *T* ≈ *L*/(0.25 ∗ 128) with 128 samples frame length and 75% overlap between each frame. Five basis vectors were obtained for each *envelopegram*. The *envelopegram* is factorized by the NMF into the basis and component matrices.

**Figure 2.**
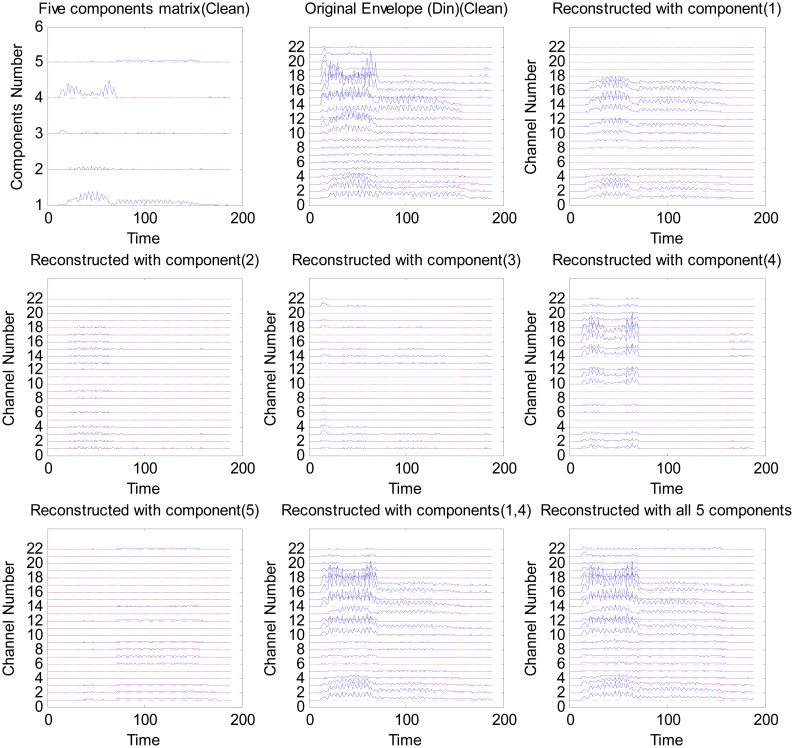
An example of the NMF reconstruction *envelopegram* with different components of the word ‘Din’ (see [39] for more detail). The top left panel is the component matrix, **W**, which determines the activation of different basis vectors over time. The top middle panel is the original *envelopegram*, **Z**, of the word ‘Din’, and the other panels are the reconstruction results with different component(s).

Figure 2 shows the reconstruction of the envelopes with different components for the word ‘Din’ processed by the sparse NMF strategy with a sparsity level of λ = 0 (no sparsification). This analysis illustrates that the representation in the NMF domain is inherently more sparse than in the time domain, indicating that NMF can reconstruct speech with reduced information by choosing only a few components. In this example, components 1 and 4 alone can reconstruct most of the envelope information (see Figure 2 bottom middle panel). This reflects that speech has a high degree of redundancy and only a few components are necessary to reconstruct an intelligible speech signal, as also shown in [16,49]. In this paper, the sparsity and the amount of information in the reconstructed signal is controlled by λ (refer to [39] for more details).

The above application of sparse NMF can be interpreted by assuming that the smaller NMF components correspond either to noise basis vectors or that they do not contribute significantly to the intelligibility of speech. By applying a sparseness constraint to the factorization, the small NMF components will be removed, and hence, a more sparse signal will be obtained, while effectively performing noise reduction and reducing redundancy. The amount of information to be removed can be controlled by tuning the sparsity λ. Ideally, λ should be SNR-dependent, as was also shown in [38,39].

## 3. Implementation of Sparse NMF Strategy on xPC Target Machine

### 3.1. Software and Hardware

**Figure 3.**
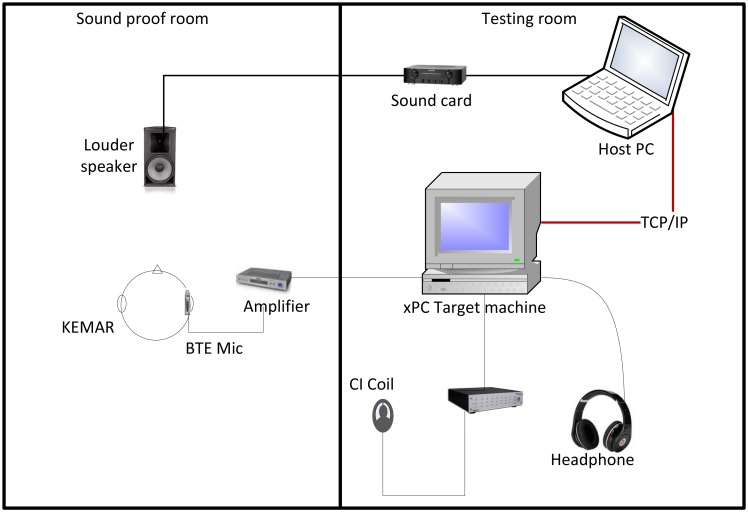
The experimental setup, including the overall architecture of the host-target HIL real-time cochlear implant (CI**)** stimulation system. The left and right parts of the figure correspond to the the sound proof room and testing room, respectively.

Figure 3 shows the overall architecture of the host-target HIL real-time CI stimulation in the experimental system.

The host PC in our case is operated by Microsoft^®^ Windows XP^®^ and runs the required software packages: MATLAB, SIMULINK**,** SIMULINK Coder, xPC Target and a C/C++ compiler (Visual C++ 2008 was used for this study). MATLAB is the host software environment of SIMULINK, SIMULINK Coder and xPC Target. SIMULINK is used to model the CI signal processor and stimulus generator. The target PC runs the highly optimized xPC Target kernel loaded from a boot disk created in MATLAB on the host PC. The communication between the host PC and the target PC is connected through a network cable and is based on the Transmission Control Protocol (TCP) and the Internet Protocol (IP) (TCP/IP protocol). SIMULINK Coder and the C/C++ compiler translate the SIMULINK model into executable code and build a target application, which is then downloaded and executed in real-time on the target PC.

For this study, an audio real time target machine (Speedgoat, Switzerland) was used. This system was bought off the shelf and provides high speed computation optimized for MathWorks SIMULINK and xPC Target. It contains a performance real-time target machine, along with high performance analog I/O ports through an XLR panel. The audio I/O modules' interface consists of twelve high-resolution 24-bit sigma-delta differential analog input and eight 16-bit differential analog output channels, which are accessed via balanced XLR connectors. Two input and one output channels were used in our experiments. During execution, the signal was routed from the analog inputs to the Target PC to be processed (see Figure 1), and then, depending on the nature of the experiment, the processed signal was either sent to the CI stimulus generator or to the analogue output channels for CI simulation.

### 3.2. Implementation of Sparse NMF S-Function

MATLAB S-functions were used for implementation of the sparse NMF algorithm. The S-function is a computer language description of a SIMULINK block written in MATLAB, C, C++ or FORTRAN. S-functions use a special calling syntax that enables the user to interact with the SIMULINK engine. This interaction is similar to the interaction that takes place between the engine and built-in SIMULINK blocks [50]. In our project, a C-MEX (MATLAB executable) S-function was developed because of its programming flexibility and stability. The primary goal of our simulation was to adapt the sparsity level λ on-line in order to measure users' individual preferences. In order to do so, the S-function parameters must be updated in real time, and hence, tuning of the parameters of the S-function needs to be enabled. During simulation (*i.e.*, execution in SIMULINK), this can be solved either by setting the parameters to be tunable or registering them as run-time parameters [50]. In external mode (*i.e.*, when executing the program on a Target machine), a Target Language Compiler (TLC) file was used to inline the S-function [50]. In our implementation, this parameter was passed as an input to the S-function, and a subsystem mask was used. By creating a subsystem mask and using a slider gain block, the user was able to adapt the value of the sparsity parameter λ in real time during the experiments without perceivable latency. Additional tunable parameters in this system involved: the range between the minimum and maximum λ, the stepsize of the NMF update rule, the NMF component number (*K*) and the NMF maximum iterative number. In our experiment, the λ range was restricted to [0,3], which was determined in pilot experiments. This range might need to be adjusted for applications with CI users, and we expect that CI users will have larger variances and will prefer larger λ values than normal listeners, but this needs to be investigated in more detail in future research.

## 4. Sparseness Parameter Tuning Experiment with xPC Target

Vocoder simulations have been widely used as a valuable tool in CI research to simulate the perception of a CI user in experiments using NH participants [6,47,51]. In vocoder studies, the signal of a CI is simulated by reconstructing an acoustical signal based on the spectral envelope [47]. Although the simulations cannot model individual CI users' performance perfectly, it has been shown, that these simulations are a good model for real CI perception, specifically for speech perception, predicting the pattern and trends in performance observed in CI users [6]. In the current study, a 12-channel noise vocoder was used.

Hu *et al.* used a two-step sparsity level selection procedure for the sparsity parameter λ [38] and found that both the normalized covariance metric (NCM) [52,53] and the short-time objective intelligibility (STOI) [54] measures can predict the intelligibility of vocoded speech to some extent. The NCM measure is similar to the speech transmission index (STI) and is a widely used measure of speech intelligibility [55]. It is based on the covariance between the input and output envelope signals and correlates highly with the intelligibility of vocoded speech, due to the similarities in the NCM calculation and CI processing strategies, that is, both of them use information extracted from the envelopes of a number of frequency bands, while discarding fine-structure information [53,56].

The computation of the NCM measure is described in detail in [52]. Briefly, the stimuli are first bandpass filtered into *Q* bands spanning the signal bandwidth, which was 8 kHz in our study (*Q* = 20 in this paper), then the envelope of each band is computed using the Hilbert transform and downsampled to 25 Hz. The SNR in each band (*SNR_i_*) is computed from the normalized covariance in the corresponding band and, subsequently, limited to the range of [–15,15] dB (refer to [52] for more detail). The transmission index (TI) in each band (*TI_i_*) is computed by linearly mapping the SNR values between zero and one using the following equation [52]:

(2) $$TI_{i}=\frac{SNR_{i}+15}{30}$$

Finally, the transmission indices are averaged across all frequency bands to produce the *NCM* index as follows:

(3) $$\text{NCM}=\frac{\sum_{1}^{Q}W_{i}\times Tl_{i}}{\sum_{1}^{Q}W_{i}}$$

where *W_i_* are the weights applied to each of the *Q* bands. The weights of each channel are listed in Table 1 (more details are in [52]).

**Figure 4.**
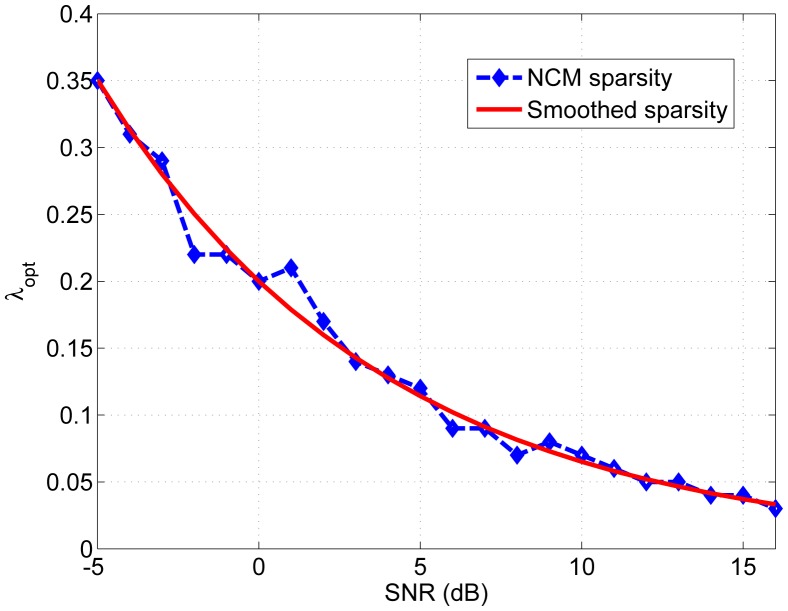
Plot of the optimum lambda values as a function of the signal-to-noise ratio (SNR) value. The blue dashed line shows the optimum values obtained by calculating the NCM of vocoded speech samples after being processed according to the sparse NMF strategy, using the Bamford-Kowal-Bench (BKB) database. The red solid curve corresponds to the fitted optimum λ values based on an exponential decay function.

Figure 4 shows the optimized λ for different SNR conditions, according to NCM. The SNR is in the range of —5 to 16 dB with 1 dB stepsize; the ‘optimized’ λ for each SNR condition is the one that achieves the highest NCM value when λ changed from 0 to 0.38, with 0.01 stepsize, denoted as λ ε [0:0.01:0.38]. In order to find the ‘optimized’ objective λ in terms of NCM, firstly, the *envelopgrams* of different SNR conditions were processed by sparse NMF with all the possible values between [0 0.38]; secondly, the noise vocoded speech signals were reconstructed based on the NMF processed *envelopgrams* of different sparsity levels λ; thirdly, all the NCM values of these vocoded speech signals were calculated and compared to each other; the maximum NCM value was found, and the corresponding λ value in each condition was selected as the optimized λ. When measuring speech intelligibility with noise vocoded speech with normal hearing subjects, we demonstrated in previous work [38] a high correlation between the NCM and λ. The blue dashed curve corresponds to the optimum λ values obtained by calculating the NCM of vocoded speech, using the Bamford-Kowal-Bench (BKB) sentences database [57]. The red solid curve shows the fitted optimum λ values as a function of the SNR value. The fitting is based on an exponential decay function, and the approximation least-squares solution is given by *λ_opt_*(*ρ*) = 0.2 · *e*^−0.1122·^*^ρ^*, where *ρ* is the SNR in dB.

Figure 4 shows that the optimized λ depends on the SNR condition, and in particular, it decreases as the SNR increases. This supports the hypothesis that a further improvement of the sparse NMF algorithm might be achieved by introducing an SNR-dependent sparsity constraint [38].

Since the optimized λ is SNR-dependent according to objective measures, this study aims to test whether this relation holds for subjective perception, as well. Thus, an on-line tuning experiment was designed using the real-time xPC Target system to test the effect of different sparsity levels. All experiments were performed in the rooms shown in Figure 3 with all sound stimuli presented through a pair of Sennheiser HDA 200 (a closed dynamic ear protector headphone designed for use with audiometers). Eight-speaker babble noise was added to the speech material at different long-term SNR conditions. All experiments were approved by the Human Experimentation Safety and Ethics Committee, Institute of Sound and Vibration Research, University of Southampton, UK.

### 4.1. Experimental Setup

Figure 3 presents the experimental setup of both rooms used for the experiments. The test data were vocoded [47] acoustical signals calculated with a 12-channel noise vocoder [6]. All sound stimuli were played at a 16 kHz sampling frequency through a Behringer UCA202 sound card and a Creek OBH-21SE amplifier connected to a PC. The sound card routed the signal through the wall of the sound attenuated room and, then, directly to the loudspeaker. The sound level of all presented samples was set to 65 dB sound pressure level (SPL). The signal was then picked up by a behind-the-ear (BTE) microphone, which was sitting on the ear of a manikin head 1.3 m away from the source. The microphone used was a Cochlear™ Nucleus^®^ SP15 dual microphone array, which was housed in a BTE shell. The microphone shell was connected to a pre-amplifier with two monophonic outputs, which were routed to two separate input ports of the xPC Target machine. The xPC Target machine processed the signal using a SIMULINK model, which was controlled by a second computer. Finally, the processed stimuli were routed to one of the xPC Target machine's output ports and were presented to the participants through the Sennheiser HDA 200 circumaural headphones.

The speech stimuli were segments of a clean speech recording of a male British English speaker reading a newspaper with a total duration of about 20 min. The duration of each segment was one minute, and the clips were randomly allocated to each participant using a Latin square procedure. The signals were corrupted with eight talker babble noise, and nine different SNR conditions were used, ranging from 0 dB to 16 dB SNR in 2 dB steps. For each SNR condition, the same clip could be repeated as many times as the participants wished, in order to select the λ value of their preference (see Section 4.2).

### 4.2. Experimental Procedure

Our hypothesis was that there should be an inverse relationship between SNR and the preferred λ, that is, as SNR increases, the average preferred value for λ will decrease. In the experiment, the preferred sparseness value λ was determined for each individual in different noise conditions. These values were based on the subjects' general preference; in other words, for each SNR condition, the subjects were asked to indicate the λ value that yielded, according to their own judgement, the maximum speech intelligibility.

Fifteen normal hearing (2 males, 13 females and aged 22-31, (mean age = 24.9 years, standard deviation = 3.0)) native English speaking participants were recruited. All participants were students from the University of Southampton, Southampton, UK. Participants were asked to listen to monaural, continuous speech through a noise vocoder CI simulation in different noisy situations. The better hearing ear, or preferred listening ear if no better ear existed, was used for the purpose of preventing any binaural listening effects. The participants sat in front of a computer screen connected to the host PC, which was running the SIMULINK model designed specifically for the experiment, and were presented with the noisy speech stimuli through circumaural headphones, which were connected to the analogue outputs of the xPC Target machine. The λ was controlled manually via an adjustment slider, which allowed participants to adjust the amount of the sparseness of the output signal according to their own preference throughout the experiment. The slider allowed λ values to change in the range from 0 to 3, which was determined during the pilot experiments, in which none of the subjects chose a λ value larger than 3 for all SNR conditions. This range, however, might need to be adjusted for experiments or applications with CI subjects. Participants were instructed to move the slider all the way to the far-right side (λ = 3) and, then, all the way to the far-left side (λ = 0) to get an idea of the range of the sound quality. Once the participants had listened to each extreme processing condition, they could then fine-tune the parameter according to their individual perceived speech intelligibility preference. The participants were instructed to take as long as they needed to perform the task, which, on average, took no more than one minute per condition. They were instructed to adjust the slider until they found subjectively that their perceived speech intelligibility was maximized. If the participants found that there was a range of slider positions in which speech intelligibility was equal, they were asked to choose a slider position based on a general preference of which sounded best to them. Participants would indicate when a preference level was chosen, and the value was recorded by the experimenter. This procedure was performed for each of the 9 different noise conditions. This procedure was repeated twice for each condition, and an average λ was calculated at each SNR for each participant. The total time to complete the experiment was around 60 min for each participant, including a 15-min break.

### 4.3. Results

**Figure 5.**
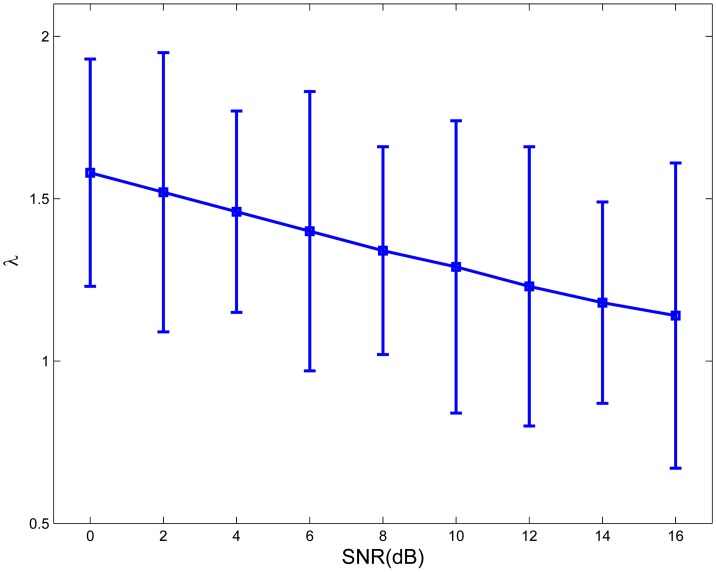
Graph of the exponential fitting of average λ values for babble noise across different SNRs.

Figure 5 shows the exponential fitting of the average λ values and the corresponding standard deviation according to the participants' subjective perceived intelligibility preferences (λ*_subjective_*) in relation to the SNR value in babble noise. The motivation for choosing an exponential fitting model was that for low-noise conditions (high SNRs), the λ value should be relatively fixed [38]. The exponential fitting function on the average experimental λ values was λ*_subjective_*(*ρ*) = 1.586 · e^−0021^*^ρ^*. A linear regression and a repeated measures ANOVA analysis were performed on the raw measured λ values of a total of 135 conditions, nine SNRs and 15 subjects, which demonstrated that SNR explained a significant proportion of the variance in λ preference values, R^2^ = 0.083, F(1,134) = 12.02, (*p* < 0.001). A significant inverse relationship between SNR and λ was found for babble noise interference, but the correlation was weak. The reason for this is presumably individual personal preference. The relationship between the values of λ and SNR was similar to the one observed in Figure 4 and in [38], which demonstrate an optimal λ calculation for different SNRs using NCM. The differences in the parameter values for the two different cases examined here (objective and subjective measures of speech intelligibility) might be due to several factors, such as different normalizing factors in the real-time implementation, the real-time test environment and individual differences.

The subjective experimental method for determining the value of λ is a novel approach for determining a sparsity parameter in speech enhancement strategies. However, more work needs to be done to establish how good participants really are at accurately determining a subjective level of sparseness to maximize their speech intelligibility. This remains to be tested in the future through a systematic speech perception experiment by comparing the performance of the sparse NMF with subjective individualized (according to individual preferences) and objective optimized (according to objective measures) λ values.

## 5. Discussions and Conclusions

Most signal processing algorithms with fixed parameters normally work for certain situations, but fail in different scenarios, so the performance is likely to be improved when these algorithms are adjusted to acoustical conditions and/or individual characteristics. In this paper, we hypothesized that listeners in general would prefer different settings for different listening conditions (specifically, different SNR) and not all listeners would choose the same settings for any given listening condition. In order to test this hypothesis, a prototyped real-time sparse NMF strategy for CIs has been developed and implemented in this paper, with various software packages (MATLAB, SIMULINK, xPC Target, SIMULINK Coder) and commercially available hardware (audio real-time target machine). By using the xPC Target along with the supported hardware and CI research platform, a sparseness constrained NMF module was implemented and embedded into the CI signal processing path. The potential benefit of a system that allows the user to adjust the processing according to the participants' individual listening needs was explored in the testing. In the listening experiment, the sparsity parameter was tuned in real-time individually at a particular time and according to their individual hearing capabilities. Results show that there is an inverse relationship between the value of λ and the value of SNR. In the future, we aim to integrate a real-time SNR estimation module into our algorithms to automatically adapt λ according to the estimated SNR. The advantage of a real-time processing system over the traditional off-line experiments conducted with CI users is that there is no need for generating and saving fixed stimuli sequences for each participant and each condition in advance. With a real-time system, it is possible to explore real-world effects with high ecological validity. While the current experiments only tested normal hearing subjects with vocoder simulations, in the future, these experiments must be extended to CI users. We expect to see more individual variation in the preferred λ values for different CI users than for normal hearing listeners. We also expect that the performance of our algorithm can be further improved by adapting the sparsity level according to the individual preference of CI users and the environmental conditions.

## Figures and Tables

**Table 1.** The weights *W_i_* applied to each of the *Q* bands (Ch) in the calculation of the normalized covariance metric (NCM) [52].

**Ch**	**W***_i_*	**Ch**	**W***_i_*	**Ch**	**W***_i_*	**Ch**	**W***_i_*	**Ch**	**W***_i_*
1	0.0772	5	0.0734	9	0.0460	13	0.0488	17	0.0520
2	0.0955	6	0.0659	10	0.0440	14	0.0488	18	0.0549
3	0.1016	7	0.0580	11	0.0445	15	0.0493	19	0.0555
4	0.0908	8	0.0500	12	0.0482	16	0.0491	20	0.0514
